# Improved efficacy against malignant brain tumors with EGFRwt/EGFRvIII targeting immunotoxin and checkpoint inhibitor combinations

**DOI:** 10.1186/s40425-019-0614-0

**Published:** 2019-05-29

**Authors:** Vidyalakshmi Chandramohan, Xuhui Bao, Xin Yu, Scott Parker, Charlotte McDowall, Yen-Rei Yu, Patrick Healy, Annick Desjardins, Michael D. Gunn, Matthias Gromeier, Smita K. Nair, Ira H. Pastan, Darell D. Bigner

**Affiliations:** 1 Department of Neurosurgery and the Preston Robert Tisch Brain Tumor Center, Duke University Medical Center, Medical Sciences Research Building, Rm 181c, Box 3156, Durham, NC 27710 USA; 2 Department of Surgery, Duke University Medical Center, Durham, NC 27710 USA; 3 Department of Medicine, Duke University Medical Center, Durham, NC 27710 USA; 4 Duke Cancer Institute Biostatistics, Duke University Medical Center, Durham, NC 27710 USA; 5 Laboratory of Molecular Biology, Center for Cancer Research, National Cancer Institute, National Institutes of Health, Bethesda, MD 20892 USA

**Keywords:** Immunotoxin, Immune checkpoint inhibitors, EGFR, T cells, Malignant gliomas

## Abstract

**Background:**

D2C7-IT is a novel immunotoxin (IT) targeting wild-type epidermal growth factor receptor (EGFRwt) and mutant EGFR variant III (EGFRvIII) proteins in glioblastoma. In addition to inherent tumoricidal activity, immunotoxins induce secondary immune responses through the activation of T cells. However, glioblastoma-induced immune suppression is a major obstacle to an effective and durable immunotoxin-mediated antitumor response. We hypothesized that D2C7-IT-induced immune response could be effectively augmented in combination with αCTLA-4/αPD-1/αPD-L1 therapies in murine models of glioma.

**Methods:**

To study this, we overexpressed the D2C7-IT antigen, murine EGFRvIII (dmEGFRvIII), in established glioma lines, CT-2A and SMA560. The reactivity and therapeutic efficacy of D2C7-IT against CT-2A-dmEGFRvIII and SMA560-dmEGFRvIII cells was determined by flow cytometry and in vitro cytotoxicity assays, respectively. Antitumor efficacy of D2C7-IT was examined in immunocompetent, intracranial murine glioma models and the role of T cells was assessed by CD4+ and CD8+ T cell depletion. In vivo efficacy of D2C7-IT/αCTLA-4/αPD-1 monotherapy or D2C7-IT+αCTLA-4/αPD-1 combination therapy was evaluated in subcutaneous unilateral and bilateral CT-2A-dmEGFRvIII glioma-bearing immunocompetent mice. Further, antitumor efficacy of D2C7-IT+αCTLA-4/αPD-1/αPD-L1/αTim-3/αLag-3/αCD73 combination therapy was evaluated in intracranial CT-2A-dmEGFRvIII and SMA560-dmEGFRvIII glioma-bearing mice. Pairwise differences in survival curves were assessed using the generalized Wilcoxon test.

**Results:**

D2C7-IT effectively killed CT-2A-dmEGFRvIII (IC_50_ = 0.47 ng/mL) and SMA560-dmEGFRvIII (IC_50_ = 1.05 ng/mL) cells in vitro. Treatment of intracranial CT-2A-dmEGFRvIII and SMA560-dmEGFRvIII tumors with D2C7-IT prolonged survival (*P* = 0.0188 and *P* = 0.0057, respectively), which was significantly reduced by the depletion of CD4+ and CD8+ T cells. To augment antitumor immune responses, we combined D2C7-IT with αCTLA-4/αPD-1 in an in vivo subcutaneous CT-2A-dmEGFRvIII model. Tumor-bearing mice exhibited complete tumor regressions (4/10 in D2C7-IT+αCTLA-4 and 5/10 in D2C7-IT+αPD-1 treatment groups), and combination therapy-induced systemic antitumor response was effective against both dmEGFRvIII-positive and dmEGFRvIII-negative CT-2A tumors. In a subcutaneous bilateral CT-2A-dmEGFRvIII model, D2C7-IT+αCTLA-4/αPD-1 combination therapies showed dramatic regression of the treated tumors and measurable regression of untreated tumors. Notably, in CT-2A-dmEGFRvIII and SMA560-dmEGFRvIII intracranial glioma models, D2C7-IT+αPD-1/αPD-L1 combinations improved survival, and in selected cases generated cures and protection against tumor re-challenge.

**Conclusions:**

These data support the development of D2C7-IT and immune checkpoint blockade combinations for patients with malignant glioma.

**Electronic supplementary material:**

The online version of this article (10.1186/s40425-019-0614-0) contains supplementary material, which is available to authorized users.

## Background

The World Health Organization (WHO) has classified gliomas into the following subtypes by histopathological features and genetic characteristics: low-grade astrocytoma, oligodendroglioma, anaplastic oligodendroglioma, anaplastic astrocytoma, and glioblastoma [1]. Glioblastoma is the most frequent and most malignant primary brain tumor in adults. It is composed of highly malignant cells that exhibit widespread infiltration into both adjacent and distant normal brain region, making the tumor highly therapy-resistant [1]. The median survival (MS) of glioblastoma patients treated with the current standard of care, including maximum safe surgical resection, radiotherapy, and concomitant chemotherapy with temozolomide, is 15–18 months [2–5]. Although currently approved therapies have led to a small improvement in glioblastoma patient survival, this improvement is plagued by systemic tissue toxicities and a poor health-related quality of life. Hence, there is a dire need for the development of new therapeutics that are capable of inducing tumor-specific durable responses with reduced morbidity, while preserving neurologic functions in glioblastoma patients.

D2C7 is a novel monoclonal antibody (mAb) that reacts with both the wild-type epidermal growth factor receptor (EGFRwt) and the mutant EGFR variant III (EGFRvIII) (both of which are major glioblastoma driver oncogenes) overexpressed on the surface of cancer cells [6, 7]. We have previously demonstrated the homogeneous reactivity of D2C7 mAb in adult glioblastoma patient samples (*n* = 101) by immunohistochemistry staining [7]. Our analysis demonstrated the positive reactivity of D2C7 mAb in virtually all cells in 100% (50/50) of the samples with *EGFRwt* amplification and in 76% (39/51) of the cases without *EGFRwt* amplification [7]. We have developed a novel cytotoxic therapeutic from D2C7 mAb for the treatment of glioblastoma; a recombinant immunotoxin, D2C7-(scdsFv)-PE38KDEL (D2C7-IT) [8]. D2C7-(scdsFv)-PE38KDEL is a genetically engineered single-chain variable-region antibody fragment (scdsFv), fused to the bacterial toxin, *Pseudomonas* exotoxin A (PE38KDEL). Upon binding to its antigen, D2C7-IT is internalized by receptor-mediated endocytosis. Once internalized, the catalytically active domain of *Pseudomonas* exotoxin A promotes the adenosine diphosphate-ribosylation and inactivation of elongation factor 2, which leads to inhibition of protein synthesis followed by cell death [9]. In preclinical studies, the dual-specific immunotoxin D2C7-IT demonstrated a strong antitumor response against intracranial glioblastoma xenografts expressing EGFRwt only and both EGFRwt and EGFRvIII, resulting in a highly significant increase in survival in the tumor-bearing animals with no viable tumor cells at the termination of the experiment. D2C7-IT is currently being evaluated in a dose-escalation phase I clinical trial for recurrent WHO grade III and IV malignant glioma patients (NCT02303678).

In addition to specifically targeting and killing tumor cells, therapies that are capable of stimulating immune responses are critical for providing long-term protection from tumor recurrence. The involvement of T cells in immunotoxin induced secondary immune responses in subcutaneous tumor models have been previously demonstrated [10, 11]. However, generation of durable antitumor immune responses by tumor-targeted cytotoxic therapies, such as D2C7-IT, is hindered by systemic and local immunosuppression in glioblastoma through the secretion of soluble factors, expression of inhibitory receptors, activation of immunosuppressive regulatory T cells (Tregs), and dysfunction/exhaustion of tumor-reactive T cells [12–19]. In addition, the immune system is also equipped with inhibitory receptors or immune checkpoints that are required for maintenance of self-tolerance and modulation of the strength of the immune response under physiological conditions. Tumors use immune checkpoint pathways to inhibit tumor-reactive T cells as a mechanism of immune resistance. Accordingly, immune resistance can be overcome by the use of antagonistic antibodies that block inhibitory receptor-ligand interactions and enhance antitumor immunity to generate durable clinical responses. The first immune checkpoint receptor to be clinically targeted was cytotoxic T-lymphocyte-associated protein 4 (CTLA-4), which is expressed on T cells and regulates the early stages of T cell activation [20]. Blocking CTLA-4 enhances tumor immunity in mouse models of brain tumor [21, 22]. A second immune checkpoint receptor on T cells is programmed cell death protein 1 (PD-1) which controls T cell function in peripheral tissues during inflammation and autoimmunity [20]. PD-1 limits the activity of T cells in the tumor via its ligand, programmed death-ligand 1 (PD-L1), expressed on tumor cells, making PD-1–PD-L1 interaction the major immune suppressive mechanism in tumor tissue. PD-L1 expression in gliomas is related to the tumor grade and is strongly associated with poor outcome [23–25]. In an open-label, phase III study (CheckMate 143), nivolumab (αPD-1) monotherapy compared with bevacizumab failed to improve median overall survival (OS) and objective response rate (ORR) in patients with recurrent glioblastoma; median OS and ORR was 9.8 months and 8% with nivolumab and 10 months and 23% with bevacizumab [26]. However, αPD-1 and radiotherapy/local chemotherapy combinations improved survival in mice with orthotopic gliomas [27, 28]. In addition to CTLA-4 and PD-1, exhausted T-cells exhibit increased expression of additional co-inhibitory receptors including TIM-3 (T-cell immunoglobulin and mucin-domain containing-3), LAG-3 (lymphocyte-activation gene 3), and TIGIT (T cell immunoreceptor with Ig and ITIM domains), broadening the therapeutic repertoire for T cell targeting [16, 29]. Another target for immune checkpoint blockade is CD73, an extracellular nucleotidase found on a variety of human tissue types, including lymphocytes and neurogliocytes [30]. One of its functions is to convert extracellular adenosine monophosphate into adenosine which causes, among other effects, the dysregulation of immune cell infiltration [31]. CD73 is also highly expressed on glioblastoma cells, positively correlated with EGFR expression, and its upregulation has been shown to both raise adenosine concentrations and lower the number of T cells in the glioblastoma microenvironment [30, 32]. We hypothesize that controlling and eliminating brain tumors requires targeted and effective tumor cell killing by cytotoxic agents and concurrent induction and maintenance of antitumor T cell function by blocking inhibitory receptors on T cells.

In the current work, we investigated D2C7-IT-mediated antitumor immunity in immunocompetent animal models of brain tumors. Our in vivo results indicate that CD4 and CD8 T cells are critical for D2C7-IT-induced antitumor response. We further demonstrate that the combination of D2C7-IT and immune checkpoint pathway inhibition elicit an effective and durable antitumor immune response that controls primary tumor growth and prevents tumor recurrence. Our findings support D2C7-IT and T cell inhibitory receptor antagonistic antibody combination therapy might improve the outcome of glioblastoma patients.

## Methods

### In vivo subcutaneous tumor model

C57BL/6 J mice (≈20 g, 6–8 weeks, female, The Jackson Laboratory) were anesthetized by isoflurane inhalation, and the appropriate flanks were shaved. A total of 3 × 10^6^ CT-2A-dmEGFRvIII-Luc tumor cells were prepared in 100 μL of Phosphate-buffered saline (PBS) and injected into the right flank. When the right tumor volumes reached an average of 100 mm^3^, mice (*n* = 10) were randomized into different treatment groups based on tumor volume. A total of four doses (1.5 μg/dose, prepared in 100 μL of 0.1% PBS-mouse serum albumin [MSA]) of D2C7-IT or control P588-IT were delivered by intratumoral injections every four days. Four doses of 250 μg/dose αPD-1 antibody (Clone RMP1–14, Bio-X-Cell) or 100 μg/dose αCTLA-4 antibody (Clone UC10-4F10–11, Bio-X-Cell) were delivered by intraperitoneal injections on the same days as D2C7-IT.

Mice from the efficacy studies surviving symptom-free for > 70 days post initial tumor implantation were re-challenged on day 72. Briefly, 1 × 10^6^ antigen negative CT-2A cells were implanted on the left flank of the surviving mice. A total of five C57BL/6 J mice (6–8 weeks, female) were used as controls.

Mice from the re-challenge studies surviving symptom-free for > 120 days post initial tumor implantation were re-challenged on day 126. Briefly, 3 × 10^5^ CT-2A-dmEGFRvIII-Luc cells were implanted intracranially on the contralateral hemisphere of the surviving mice using the coordinates described below. A total of five C57BL/6 J mice (6–8 weeks, female) were used as controls.

For the bilateral tumor model, on day 0, a total of 3 × 10^6^ and 1 × 10^6^ CT-2A-dmEGFRvIII-Luc cells were injected into the right and left flank of C57BL/6 mice, respectively. When the right tumor volumes reached an average of 100 mm^3^, mice (*n* = 10) were randomized into different treatment groups and were treated every three days as described above except a higher dose of D2C7-IT (4.5 μg/dose) was used in this study to induce stronger tumor cell killing.

Tumors were measured twice weekly with a handheld Vernier caliper, and the tumor volumes were calculated in cubic millimeters by using the formula: ([length] × [width^2^])/2. Animals were tested out of the study when tumor volume was 1500–2000 mm^3^ (Additional file 2).

## Results

### Generation of immunocompetent brain tumor models for D2C7-IT therapy

Since D2C7-IT binds human EGFR but not mouse EGFR (mEGFRwt/mEGFRvIII), we engineered D2C7-IT for studies in syngeneic, immunocompetent murine models. The D2C7 epitope is composed of a conformational determinant of 55 amino acids. There is a mismatch of seven amino acids between the human (h) and mouse (m) EGFR sequence in the D2C7 epitope region (Additional file 1: Figure S1). To facilitate D2C7 binding to mEGFRvIII, the DNA sequence corresponding to the seven amino acids in the mEGFRvIII protein sequence was mutated by site-directed mutagenesis to create the human D2C7 epitope. This chimeric construct was designated D2C7 (d)-mouse (m)-EGFRvIII (dmEGFRvIII) and was used to generate brain tumor cell lines for D2C7-IT targeted therapy.

### Binding of the D2C7 mAb and cytotoxicity of D2C7-IT against mouse brain tumor cell lines

**Fig. 1:**
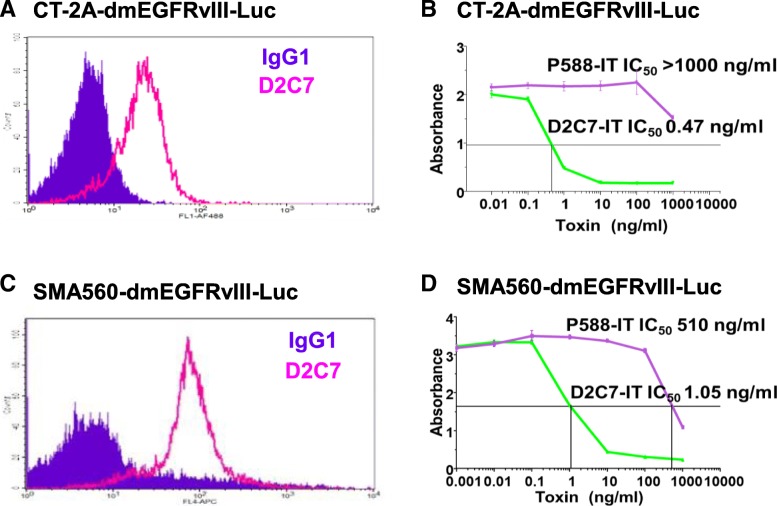
Flow cytometric analysis of D2C7 mAb and cytotoxicity of D2C7-IT against malignant glioma cell lines. **a** and **c** CT-2A-dmEGFRvIII-Luc (**a**) and SMA560-dmEGFRvIII-Luc (**c**) cell lines were stained with D2C7-AF488/−APC (pink open peaks) or IgG1-AF488/−APC isotype control Ab (filled purple peaks). **b** and **d** Cytotoxicity of D2C7-IT against CT-2A-dmEGFRvIII-Luc (**b**) and SMA560-dmEGFRvIII-Luc (**d**) cell lines was assessed by the WST-1 assay. The data are given as IC_50_ values, the concentration of D2C7-IT that causes 50% tumor cell death after a 48-h incubation

The astrocytic brain tumor cell lines CT-2A and SMA560 were transduced with dmEGFRvIII and luciferase (Luc) constructs to establish the CT-2A-dmEGFRvIII-Luc and SMA560-dmEGFRvIII-Luc cell lines. The D2C7 mAb exhibited binding to the brain tumor cell lines CT-2A-dmEGFRvIII-Luc and SMA560-dmEGFRvIII-Luc (Fig. 1a and c). The cytotoxicity (IC_50_ measurements) of D2C7-IT on the CT-2A-dmEGFRvIII-Luc and SMA560-dmEGFRvIII-Luc cells were 0.47 ng/mL and 1.05 ng/mL, respectively (Fig. 1b and d). The control immunotoxin, P588-IT, exhibited no (> 1000 ng/mL) or non-specific (510 ng/mL) cytotoxic activity against both CT-2A-dmEGFRvIII-Luc and SMA560-dmEGFRvIII-Luc cells, respectively.

### Determination of D2C7-IT maximum tolerated dose (MTD)

To determine the MTD of D2C7-IT for intracranial treatment, CT-2A-dmEGFRvIII-Luc tumor-bearing C57BL/6 immunocompetent mice were treated with 0.03, 0.1, 0.3, and 1 μg of D2C7-IT by CED for 72 h. Significant acute toxicity (4/8 mice total; Additional file 1: Figure S2) was observed with the highest dose of D2C7-IT (1 μg) after the 72-h infusion. Total doses of 0.1 μg and 0.3 μg of D2C7-IT that had no toxicity-associated mortality were chosen as the appropriate dose for further evaluation.

### Antitumor efficacy of D2C7-IT therapy in intracranial glioma models

**Fig. 2:**
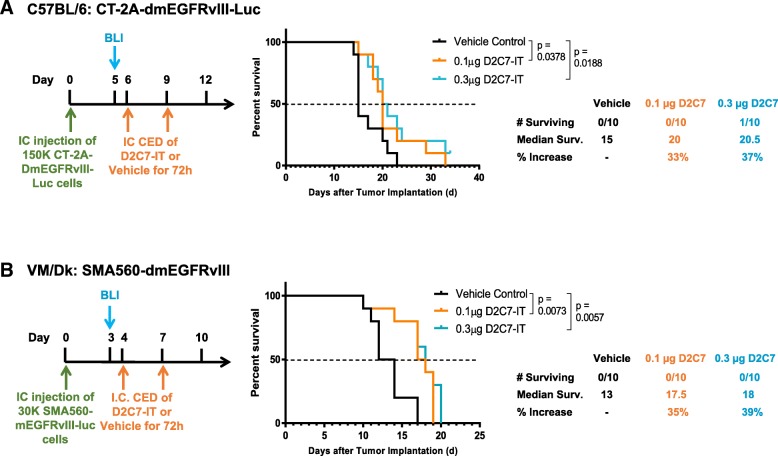
Dose-response comparison of two different D2C7-IT concentrations in orthotopic glioma models. Treatment schedule, survival curves, and median survival are shown for C57BL/6 J mice bearing intracranial CT-2A-dmEGFRvIII-Luc tumors (**a**) and VM/Dk mice bearing intracranial SMA560-dmEGFRvIII-Luc tumors (**b**) infused with either vehicle control, 0.1μg D2C7-IT, or 0.3μg D2C7-IT. The *p*-values generated from the generalized Wilcoxon test are provided for both tumor models

Orthotopic models of brain tumors were established with CT-2A-dmEGFRvIII-Luc and SMA560-dmEGFRvIII-Luc cell lines in C57BL/6 and VM/Dk immunocompetent mice, respectively. Based on MS in untreated mice, days 4 and 6 post-tumor implantations were selected as the optimal time to initiate D2C7-IT infusion by CED in SMA560-dmEGFRvIII-Luc (MS = 13) and CT-2A-dmEGFRvIII-Luc (MS = 15) tumors, respectively. Mice were examined by bioluminescence imaging (BLI) on days 3 (SMA560-dmEGFRvIII-Luc) and 5 (CT-2A-dmEGFRvIII-Luc) and were randomized into different treatment groups based on total flux. In the CT-2A-dmEGFRvIII-Luc intracranial tumor model, orthotopic delivery of 0.1 μg (*P* = 0.0378) or 0.3 μg (*P* = 0.0188) of D2C7-IT corresponded with an increase in MS of 33 and 37% compared to vehicle control, respectively (Fig. 2a). Similarly, in the SMA560-dmEGFRvIII-Luc intracranial tumor model, increases in MS of 35 and 39% were observed post 0.1 μg (*P* = 0.0073) or 0.3 μg (*P* = 0.0057) of D2C7-IT infusion compared to control, respectively (Fig. 2b). Thus, D2C7-IT generated significant improvement in survival in two different immunocompetent mouse models of brain tumors. Next, we compared the efficacy of D2C7-IT and control immunotoxin P588-IT on tumor antigen positive CT-2A-dmEGFRvIII-Luc and tumor antigen negative parental CT-2A cell lines, respectively (Additional file 1: Figure S3). In the CT-2A-dmEGFRvIII-Luc intracranial tumor model, orthotopic delivery of 0.1 μg of D2C7-IT generated a 164% increase in MS compared to vehicle control (*P* < 0.0001) (Additional file 1: Figure S3A), and at the end of the study period there were three tumor-free mice in the D2C7-IT monotherapy group. Neither tumor-free mice nor an increase in the MS was observed in control P588-IT group compared to vehicle control (Additional file 1: Figure S3A), thus eliminating the possibility of a nonspecific effect of the PE38KDEL on the tumor microenvironment. Orthotopic delivery of 0.1 μg of D2C7-IT or P588-IT failed to increase MS in the parental CT-2A intracranial tumor model, thereby demonstrating the specificity of D2C7-IT to EGFRvIII tumor antigen (Additional file 1: Figure S3B).

### Effect of CD4+ and CD8+ T cell depletion on D2C7-IT-mediated antitumor response

**Fig. 3:**
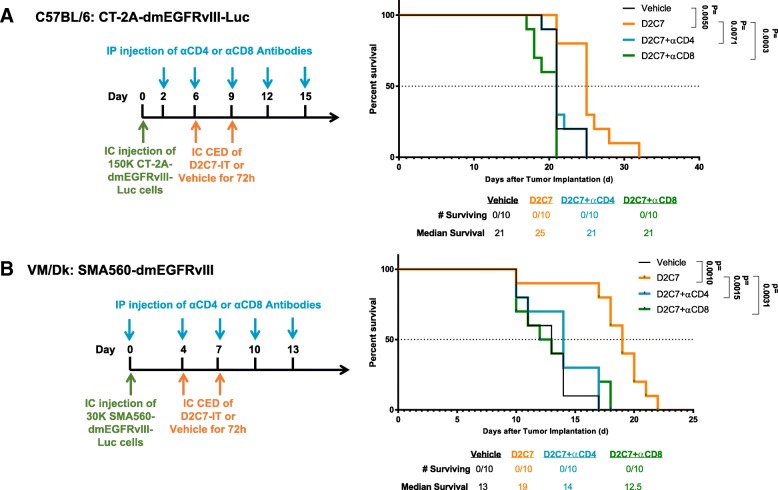
Antitumor effects of D2C7-IT therapy post-CD4+ and CD8+ T cell depletion in orthotopic glioma models. Treatment schedule, survival curves, and median survival are shown for C57BL/6 J mice bearing intracranial CT-2A-dmEGFRvIII-Luc tumors (**a**) and VM/Dk mice bearing intracranial SMA560-dmEGFRvIII-Luc tumors (**b**). The *p*-values generated from the generalized Wilcoxon test are provided for both tumor models

To determine if D2C7-IT activates secondary immune responses, we depleted T lymphocyte subsets in intracranial CT-2A-dmEGFRvIII-Luc and SMA560-dmEGFRvIII-Luc glioma-bearing mice (*n* = 10/group). D2C7-IT (0.3 μg) delivered by CED for 72 h extended MS from 21 to 25 and 13 to 19 days against CT-2A-dmEGFRvIII-Luc (Fig. 3a**)** and SMA560-dmEGFRvIII-Luc tumors (Fig. 3b), respectively. The therapeutic efficacy of D2C7-IT was completely abolished upon CD4+ or CD8+ T cell depletion in the CT-2A-dmEGFRvIII-Luc and SMA560-dmEGFRvIII-Luc models (Fig. 3). Thus, in addition to the direct cytotoxic effect on tumor cells, D2C7-IT generates a secondary CD4+ and CD8+ T cell immune response.

### PD-1 and FoxP3 expression in T cells in mouse models of brain tumors

Since T cells contributed to D2C7-IT-mediated antitumor response (Fig. 3) and glioblastomas support an immunosuppressive microenvironment [13, 14, 18, 19], we examined the phenotype of CD4+ and CD8+ T cells and tumor cells in CT-2A-dmEGFRvIII-Luc (day 6 and end-stage), and SMA560-dmEGFRvIII-Luc (day 4 and end-stage) intracranial tumors. Flow cytometry analysis demonstrated the expression of inhibitory receptor PD-1 on CD4+ and CD8+ T cells and its ligand PD-L1 on tumor cells in CT-2A-dmEGFRvIII-Luc and SMA560-dmEGFRvIII-Luc tumors (Additional file 1: Figure S4). Similarly, immunosuppressive FoxP3 + CD4+ T cells were present in both CT-2A-dmEGFRvIII-Luc and SMA560-dmEGFRvIII-Luc intracranial tumors (Additional file 1: Figure S4B and S4F). Multiplex immunofluorescence analysis showed the expression of PD-1 on CD8+ and FoxP3 on CD4+ T cells in CT-2A-dmEGFRvIII-Luc and SMA560-dmEGFRvIII-Luc tumors (Additional file 1: Figure S5A-B, left panels). PD-L1 expression on tumor cells in CT-2A-dmEGFRvIII-Luc and SMA560-dmEGFRvIII-Luc tumors (Additional file 1: Figure S5A-B, right panels) was confirmed by immunohistochemistry. Our data indicate that combining D2C7-IT with antibodies that block the immune checkpoints PD-1 (to reactivate exhausted tumor-specific T cells) and CTLA-4 (to inhibit immunosuppressive Tregs), or PD-L1 (to block PD1/PDL1 pathway) may reinstate immunosurveillance in brain tumors.

### D2C7-IT and αPD-1/αCTLA-4 combination therapy in the subcutaneous CT-2A-dmEGFRvIII-Luc model

**Fig. 4:**
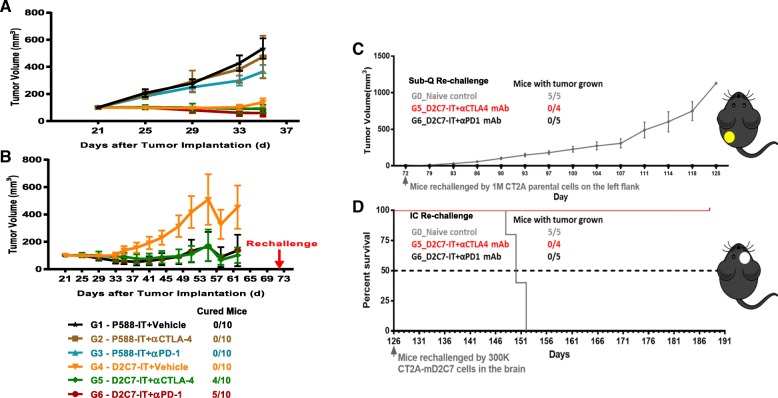
In vivo efficacy of D2C7-IT+αCTLA-4/αPD-1 combination therapies in subcutaneous CT-2A-dmEGFRvIII-Luc tumor-bearing C57BL/6 J mice. (**a**) Survival curves for groups 1–6 (G1–6) followed up to Day 35 and (**b**) Groups 4–6 (G4–6) followed up to Day 62 after initial tumor inoculation are presented as SEM. (**c**) Survival curves are presented as SEM for mice surviving symptom-free to Day 72 post-tumor implantation from different treatment groups and were re-challenged initially in the left flank with 1 × 10^6^ CT-2A parental cells. C57BL/6 J mice (*N* = 5) were used as naïve controls. (**d**) Mice surviving symptom-free from different treatment groups after first re-challenge underwent a second re-challenge with 3 × 10^5^ CT-2A-dmEGFRvIII-Luc cells in the brain on Day 126. C57BL/6 J mice (*N* = 5) were used as naïve controls. Median survival estimates are presented

The in vivo efficacy of D2C7-IT/αCTLA-4/αPD-1 monotherapy or D2C7-IT+αCTLA-4/αPD-1 combination therapy was evaluated in subcutaneous CT-2A-dmEGFRvIII-Luc bearing C57BL/6 immunocompetent mice. In the in vivo subcutaneous CT-2A-dmEGFRvIII-Luc model, four cycles of the D2C7-IT (but not αCTLA-4/αPD-1) monotherapies and D2C7-IT+αCTLA-4/αPD-1 combination therapies generated a significant delay in tumor growth compared to the control immunotoxin (P588-IT) treatment groups (Fig. 4a). Importantly, complete tumor regressions were only observed in the D2C7-IT+αCTLA-4 (*n* = 4/10) and D2C7-IT+αPD-1 (*n* = 5/10) combination therapy groups (Fig. 4b). Further, surviving mice from the D2C7-IT+αCTLA-4 (*n* = 4/10) and D2C7-IT+αPD-1 (*n* = 5/10) combination therapy groups failed to develop tumors upon subcutaneous re-challenge (day = 72) with the parental CT-2A cell line that does not express the target antigen, EGFR, indicating that the combination therapy induced broadly protective antitumor immunity (Fig. 4c). We also observed the induction of protective antitumor immunity in mice re-challenged intracranially (day = 126) with the target antigen-expressing CT-2A-dmEGFRvIII-Luc cells (Fig. 4d).

**Fig. 5:**
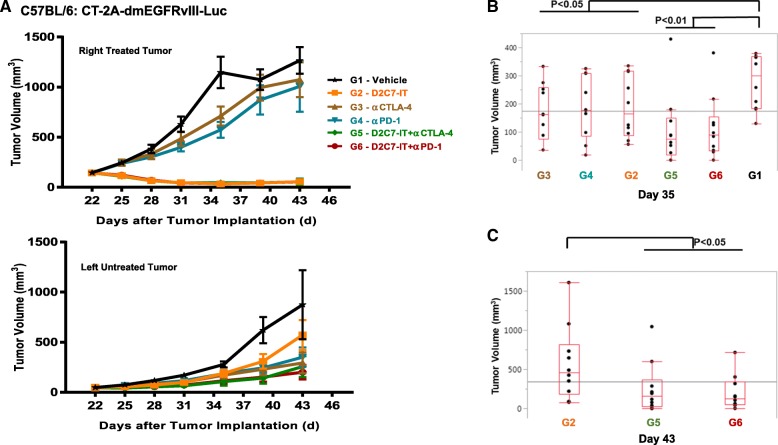
In vivo efficacy of D2C7-IT+αCTLA-4/αPD-1 combination therapies in bilateral subcutaneous CT-2A-dmEGFRvIII-Luc tumor-bearing C57BL/6 J mice. (**a**) The tumor growth curves are presented as SEM for the right treated tumors (upper panel) and left untreated tumors (lower panel). **b** and **c** Differences in left tumor volumes were assessed between the vehicle control group (G1) and all other treatment groups (G2–6) on Day 35 (**b**) and between D2C7-IT monotherapy group (G2) and D2C7-IT+αCTLA-4/αPD-1 combination therapy groups (G5–6) on Day 43 (**c**) and are not adjusted for multiple testing

We next investigated the efficacy of D2C7-IT/αCTLA-4/αPD-1 monotherapies or D2C7-IT+αCTLA-4/αPD-1 combination therapies in vivo in a bilateral subcutaneous CT-2A-dmEGFRvIII-Luc model. On day 0, a total of 3 × 10^6^ and 1 × 10^6^ CT-2A-dmEGFRvIII-Luc tumor cells were injected into the right and left flank of C57BL/6 mice, respectively. When the right tumors reached an average volume of 100 mm^3^, they were treated with four cycles of D2C7-IT/αCTLA-4/αPD-1 monotherapies or D2C7-IT+αCTLA-4/αPD-1 combination therapies, while the left tumors were left untreated. The D2C7-IT monotherapy and D2C7-IT+αCTLA-4/αPD-1 combination therapies led to significant growth delay of the right tumors (Fig. 5a). On day 35 post-tumor implantation, a significant tumor growth delay was observed in the left untreated tumors as compared to the vehicle control group in mice treated with D2C7-IT/αCTLA-4/αPD-1 monotherapies (exact Wilcoxon rank-sum *P* < 0.05) or D2C7-IT+αCTLA-4/αPD-1 combination therapies (exact Wilcoxon rank-sum *P* < 0.01) (Fig. 5b). Compared to D2C7-IT monotherapy (day 43 post-tumor implantation), a significant decrease in left tumor volume was observed with the D2C7-IT+αCTLA-4/αPD-1 combination therapies (exact Wilcoxon rank-sum *P* < 0.05) (Fig. 5c), indicating the induction of a systemic immune response.

### D2C7-IT and αPD-1/αCTLA-4 combination therapies in the intracranial CT-2A-dmEGFRvIII-Luc glioma model

**Fig. 6:**
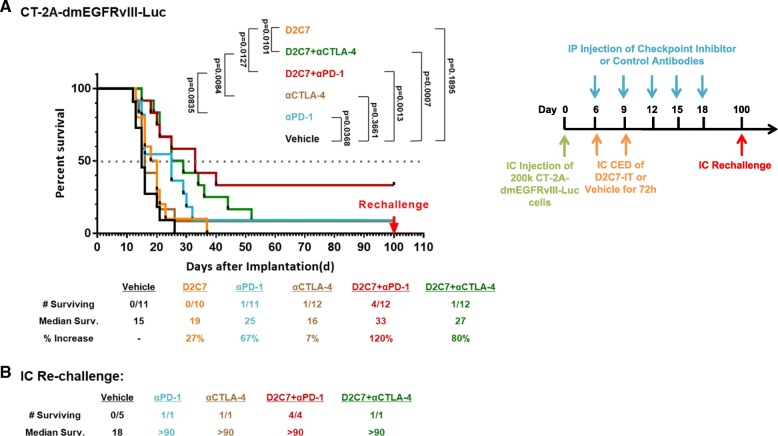
Treatment of CT2A-dmEGFRvIII-Luc intracranial tumors with D2C7-IT and αPD-1/αCTLA-4 combinations. (**a**) Survival curves and median survival estimates for CT2A-dmEGFRvIII-Luc tumor-bearing mice treated with vehicle control, D2C7-IT, αPD-1, and αCTLA-4 mono or combination therapies. The p-values were generated from the generalized Wilcoxon test and are not adjusted for multiple testing. (**b**) Median survival was estimated for mice surviving symptom-free to Day 100 post-tumor implantation from different treatment groups and were re-challenged in the contralateral hemisphere of the brain with 1.5 × 10^5^ CT-2A-dmEGFRvIII-Luc cells. C57BL/6 J mice (*N* = 5) were used as naïve controls

Since our subcutaneous studies indicated improved survival with or D2C7-IT+αCTLA-4/αPD-1 combination therapies (Figs. 4 and 5), we next evaluated their efficacy in intracranial CT-2A-dmEGFRvIII-Luc glioma-bearing C57BL/6 mice. In comparison to the vehicle control treatment group, we observed a ≈ 30–60% increase in MS with the D2C7-IT/αPD-1 monotherapies, an 80% increase in MS with the D2C7-IT+αCTLA-4 combination therapy, and a 120% increase in MS with the D2C7-IT+αPD-1 combination therapy (Fig. 6a). At the end of the study period, there was one mouse each that was tumor free in the αCTLA-4/αPD-1 monotherapy groups (Fig. 6a), and there were 4 mice and 1 mouse that were tumor free in the D2C7-IT+αPD-1 (Fig. 6a) and D2C7-IT+αCTLA-4 (Fig. 6a), combination therapy groups, respectively. Surviving mice from both the monotherapy and combination therapy groups were re-challenged intracranially on day 60 with CT-2A-dmEGFRvIII-Luc cell line (Fig. 6b). While all the control mice developed tumors, all of the surviving mice from either the monotherapy or the combination therapy groups resisted tumor re-challenge (Fig. 6b), indicative of the generation of a systemic antitumor immune response.

### Antitumor efficacy of D2C7-IT and αPD-1/αPD-L1/αTim-3/αLag-3/αCD73 combination therapies against intracranial gliomas

**Fig. 7:**
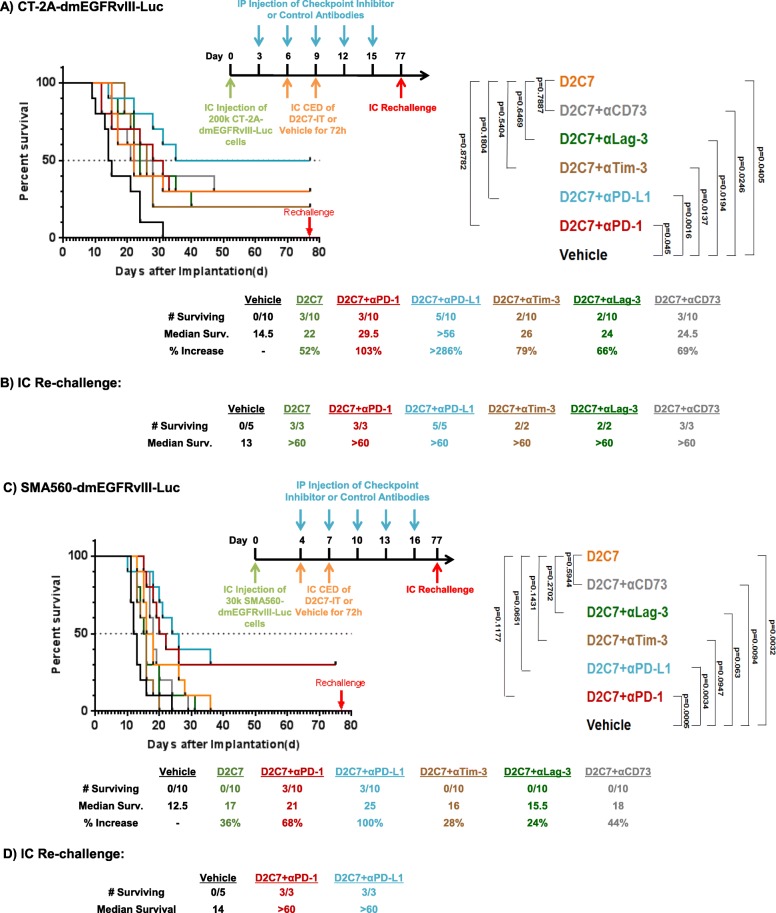
Anti-tumor efficacy of D2C7-IT and immune checkpoint inhibitor combinations in orthotopic glioma models. **a** and **c** Survival curves and median survival estimates data are shown for C57BL/6 J mice bearing intracranial CT-2A-dmEGFRvIII-Luc tumors (**a**) and VM/Dk mice bearing intracranial SMA560-dmEGFRvIII-Luc tumors (**c**) treated with vehicle control, D2C7-IT monotherapy, or D2C7-IT+αPD-1/αPD-L1/αTim-3/αLag-3/αCD73 combinations. The p-values generated from the generalized Wilcoxon test are provided for both tumor models and are not adjusted for multiple testing. **b** and **d** Median survival for mice that survived to Day 77, and were re-challenged in the contralateral hemisphere of the brain with 2 × 10^5^ CT-2A (**b**), or 3 × 10^4^ SMA560 (**d**) parental cells. C57BL/6 J mice (*N* = 5) or VM/Dk (*N* = 5) were used as naïve controls

Since our initial intracranial study (Fig. 6a) demonstrated a significant increase in survival when D2C7-IT was used in combination with αPD-1 and αCTLA-4 antibodies, and glioblastomas are known to express additional checkpoint inhibitors including PD-L1/Tim-3/Lag-3/CD73 [16, 23, 32] we extended our analysis to include blockade of these immune checkpoint inhibitors as possible therapeutic agents. In comparison to the vehicle control group, we observed a ≈ 52–100% increase in MS with the D2C7-IT monotherapy and D2C7-IT+αPD-1/αTim-3/αLag-3/αCD73 combination therapies, and a > 286% increase in MS with the D2C7-IT+αPD-L1 combination therapy (Fig. 7a). At the end of the study there were tumor-free mice in the D2C7-IT monotherapy and different combination therapy groups, with the maximum numbers observed in the D2C7-IT+αPD-L1 combination therapy group (Fig. 7a). On day 77 post-tumor implantation all surviving mice were intracranially re-challenged with target antigen-negative 2 × 10^5^ CT-2A parental line, along with five naïve controls (Fig. 7b). All of the naïve control mice developed tumors and failed to survive the re-challenge, while none of the mice from the D2C7-IT monotherapy or D2C7-IT+immune checkpoint inhibitor combination developed tumors (Fig. 7b).

The D2C7-IT+αPD-1/αPD-L1/αTim-3/αLag-3/αCD73 antibody combination therapy study in the SMA560-dmEGFRvIII-Luc glioma model generated a ≈ 24–100% increase in the MS of the D2C7-IT monotherapy and the different combination therapy groups (Fig. 7c). Three mice remained alive in each of the D2C7-IT+αPD-1 and D2C7-IT+αPD-L1 combination therapy groups at the end of the study (Fig. 7c). These mice, in addition to five naïve controls, were intracranially re-challenged on day 77 with 3 × 10^4^ SMA560 parental line (Fig. 7d). All five naïve control mice succumbed to tumors, while all the mice from the D2C7-IT+αPD-1 and D2C7-IT+αPD-L1 combination therapy groups remained tumor free (Fig. 7d).

Additional experiments were performed to compare the efficacy of the D2C7-IT/αPD-L1/αTim-3/αLag-3/αCD73 monotherapies and D2C7-IT+αPD-L1/αTim-3/αLag-3/αCD73 combination therapies (Additional file 1: Figure S6). We observed a 142% increase in MS with the D2C7-IT monotherapy and a 232% increase with both αPD-L1 monotherapy and D2C7-IT+αPD-L1 combination therapy (Additional file 1: Figure S6A). At the end of the study, there were 4–5 alive mice in the D2C7-IT or αPD-L1 monotherapy groups and 8/8 alive mice in the D2C7-IT+αPD-L1 combination therapy group (Additional file 1: Figure S6A). In comparison to the D2C7-IT monotherapy, we did not observe significant improvement in survival with αTim-3/αLag-3/αCD73 monotherapies and D2C7-IT+αTim-3/αLag-3/αCD73 combination therapies (Additional file 1: Figure S6B-D).

## Discussion

Glioblastomas are highly heterogeneous, inherently aggressive, and immunosuppressive tumors that evade the host immune system resulting in suboptimal responses to immunotherapy. D2C7-IT is a cytotoxic agent that targets both EGFRwt and mutant EGFRvIII. In this study, we investigated the mechanism of D2C7-IT-induced antitumor response. Our results indicate that in addition to eliminating tumor cells in a targeted manner, D2C7-IT induce T cell responses as evidenced by the fact that depletion of T cells reduces IT-mediated antitumor response. Immune checkpoint therapies that block inhibitory receptors on T cells are used to overcome tumor immune suppression and improve treatment outcome for patients with melanoma and lung cancer, but not glioblastoma [26, 33–35]. Interestingly, checkpoint inhibitors only benefit a small subset of patients, specifically those who have a pre-existing antitumor immune response [36]. We, therefore, investigated if the antitumor efficacy of D2C7-IT can be augmented with immune checkpoint blockade in murine glioma models. Our findings indicate that D2C7-IT in combination with PD-1/PD-L1 blockade elicit targeted tumor cell cytotoxicity and an effective and durable adaptive T cell response.

We used two immunocompetent murine malignant glioma models CT-2A-dmEGFRvIII-Luc and SMA560-dmEGFRvIII-Luc, for the assessment of D2C7-IT and immune checkpoint inhibitor combination efficacy. In intracranial CT-2A-dmEGFRvIII-Luc and SMA560-dmEGFRvIII-Luc tumor models, orthotopic delivery of D2C7-IT generated significant improvement in survival (*P* < 0.05) (Fig. 2). Further, depletion studies demonstrated complete abrogation of D2C7-IT specific antitumor responses in the absence of CD4+ or CD8+ T cells (Fig. 3). Our data suggest that the remarkable response observed in phase I studies with the ITs, TP-38 (patient surviving > 5 years post-therapy) [37] and IL13-PE38QQR (prolonged progression-free survival beyond one and two years in 15/46 glioblastoma patients) [38] could be attributed to IT-mediated induction of T cell immune response. In the ongoing phase I clinical trial (NCT02303678), 36 patients have been treated with D2C7-IT. Encouraging efficacy results are observed, with fourteen patients remaining alive and one patient continuing to persist disease-free > 29.4 months after D2C7-IT infusion [39], suggesting the involvement of T cell response.

Since inhibitory receptors are known to dampen immune response in malignant brain tumors [16], we examined the presence of such receptors on T cells in intracranial tumors. Examination of CT-2A-dmEGFRvIII-Luc and SMA560-dmEGFRvIII-Luc tumors established the expression of inhibitory receptors PD-1 and FoxP3 on T cells and inhibitory ligand PD-L1 on tumor cells. This data led us to examine the efficacy of D2C7-IT and immune checkpoint inhibitor combinations in the murine glioma models. Due to the complexity of intracranial tumor models involving multiple treatment groups (≥6) and size (*n* = 10/group) we were interested in conducting the initial antitumor efficacy studies in subcutaneous glioma models and therefore examined the immune cell composition in subcutaneous and intracranial tumors by flow cytometry. Except for microglia, flow cytometry analysis revealed similar immune cell composition in subcutaneous and intracranial glioma models. Therefore we chose to pursue the preliminary antitumor efficacy assessment of D2C7-IT and immune checkpoint inhibitor combinations in subcutaneous murine glioma models and subsequently validated the results in intracranial tumor models. In the subcutaneous CT-2A-dmEGFRvIII-Luc model significant tumor growth delays and cures were observed specifically in the combination therapy groups (Fig. 4). Additionally, subcutaneous and intracranial re-challenge of surviving tumor-free mice demonstrated the induction of a systemic immune response against CT-2A/CT-2A-dmEGFRvIII-Luc tumors post D2C7-IT+αCTLA-4/αPD-1 combination therapies (Fig. 4). Finally, intratumoral injections of D2C7-IT combined with systemic delivery of αCTLA-4/αPD-1 induced a secondary immune response and promoted regression of a distant tumor (Fig. 5). These findings indicate that the combination therapy potentiates immune activation at the local tumor milieu which then spreads systemically and eliminates tumors at distant sites.

Next, we examined the efficacy of the D2C7-IT+αCTLA-4/αPD-1 combination therapy in the intracranial CT-2A-dmEGFRvIII-Luc glioma model. Similar to our sub-cutaneous study significant tumor growth delays, cures, and resistance to tumor re-challenge were observed in the combination therapy groups (Fig. 6). Due to the incidence of severe immune-related adverse events in patients receiving αCTLA-4 antibodies in the clinic, we decided not to pursue the D2C7-IT+αCTLA-4 combination [35, 40]. We also explored the therapeutic benefit of additional checkpoint inhibitors including αPD-1/αPD-L1/αTim-3/αLag-3/αCD73 in combination with D2C7-IT. In both the CT-2A-dmEGFRvIII-Luc and SMA560-dm-EGFRvIII-Luc glioma models, the use of αPD-L1 or αPD-1 antibodies in combination with D2C7-IT proved to be the most effective in increasing MS and mice survival (Fig. 7). The other immune checkpoint inhibitors, αTim-3/αLag-3/αCD73, tested in combination with D2C7-IT proved to be less effective, failing to match or exceed D2C7-IT+αPD-L1/αPD-1 treatments both in MS increase and in the number of surviving mice. All of the surviving mice resisted tumor re-challenge with antigen-negative parental glioma cell line CT-2A indicating the generation of a broadly protective systemic antitumor immunity.

In the absence of real-time tumor visualization, in vivo efficacy of the D2C7-IT and checkpoint inhibitor combination against intracranial tumors is influenced by multiple factors including tumor cell count, tumor size, tumor location, precise delivery of the immunotoxin, and treatment regimen. Unlike subcutaneous tumor models, with the highly aggressive CT-2A-dmEGFRvIII-Luc and SMA560-dm-EGFRvIII-Luc intracranial glioma models, even a minor variability in one of the above factors would cause a significant difference in the therapeutic efficacy. Thus we would hypothesize that one or a combination of the above technical factors contributed to the variability in survival observed post-D2C7-IT and checkpoint inhibitor mono and combination therapies.

Recently it was demonstrated in both CT-2A and SMA560 intracranial tumors that PD-1 was expressed on the majority of CD8+ T-cells, and Tim-3 and Lag-3 were predominantly co-expressed with PD-1 [16]. Additionally, in CT-2A orthotopic tumors, PD-1 was shown to be present on ≈50% of CD8+ T-cells, while Tim-3 and Lag-3 were expressed on ≈2–4% of CD8+ T-cells [16]. We believe that the low expression of Tim-3 and Lag-3 in the malignant glioma models contributed to the sub-optimal efficacy of the D2C7-IT+αTim-3/αLag-3 therapies. In the absence of CD73 expression data in the CT-2A and SMA560 tumor models, we hypothesize that lower levels of expression translated into decreased therapeutic efficacy with D2C7-IT+αCD73 therapy.

For all our subcutaneous therapy studies we were able to inject a high dose of D2C7-IT (6–18 μg total dose) into tumors to achieve a therapeutic response in the absence of non-specific systemic toxicity. However, in our intracranial tumor models, we observed non-specific toxicity at a total dose of D2C7-IT > 0.3 μg. We believe that D2C7-IT dose limitation prevented us from achieving significant therapeutic benefits with this drug as a single agent. We also used a nonspecific IT, P588-IT, as a control in subcutaneous and intracranial studies. No significant therapeutic benefit from an intratumoral injection of the control IT was observed confirming that antigen-targeted tumor cell killing by D2C7-IT resulted in the generation of systemic immunity. Finally, both CT-2A-dmEGFRvIII-Luc and SMA560-dm-EGFRvIII-Luc glioma models are extremely aggressive with a MS of ≤15 days. Achieving significant improvement in MS and the number of tumor-free mice in such aggressive models with a total dose of 0.1 μg D2C7-IT+αPD-L1/αPD-1 therapy reinforces the therapeutic benefit of the combinations and provides a strong rationale for clinical translation.

Flow cytometry analysis was undertaken to unravel the role of T cells in the increased efficacy of D2C7-IT+αPD-1/αCTLA-4 therapies. Preliminary data indicated an increase in both CD4:Treg and CD8:Treg ratio in the CT-2A-dmEGFRvIII-Luc intracranial tumors specifically in the combination therapy groups (Additional file 1: Figure S7). A previous study involving anti-mesothelin IT (SS1P) and αCTLA-4 combination demonstrated a significant increase in CD8+ T cells in the mammary tumors in the combination therapy group [41]. However, we did not see an increase in the total number of CD4+ or CD8+ T cells after D2C7-IT+αPD-1/αCTLA-4 combinations. An increase in antigen-specific T cells both in the tumor and draining lymph nodes was demonstrated previously post chemotherapy and αPD-1 combinations in an intracranial tumor model with the GL261-Ova cell line [27]. We are in the process of establishing CT-2A-dmEGFRvIII-Trp2 and CT-2A-dmEGFRvIII-Ova cell lines to investigate the presence of antigen-specific T cells post D2C7-IT and checkpoint inhibitor combination therapies. Additionally, flow cytometry analysis failed to show changes in the numbers of microglia, macrophages, B cells, NK cells, and neutrophils during D2C7-IT/αPD-1/αCTLA-4 mono and combination therapies. Future studies will focus on elucidating the mechanism for the improved efficacy of the D2C7-IT+αPD-L1/αPD-1 combinations.

Finally, in phase II trials, Rindopepimut (CDX-110), a peptide vaccine targeting EGFRvIII, improved overall survival in newly diagnosed GBM patients expressing EGFRvIII [42]. However, in a randomized, double-blinded, phase III trial, despite the induction of an EGFRvIII-specific antibody response in the majority of patients, rindopepimut cohort failed to outperform the control KLH cohort [43]. Preclinical mechanistic studies showed a role for antibody-dependent cellular cytotoxicity (ADCC) in the antitumor response following EGFRvIII peptide vaccination [44]. Effective tumor cell killing by ADCC is dependent on Fc receptor-mediated activation of NK cells, granulocytes, and macrophages and this pathway could be profoundly compromised in highly immunosuppressed GBM patients, in turn, contributing to the failure of phase III rindopepimut trial. In contrast, cytotoxic agents such as D2C7-IT would potentiate direct tumor cell killing and as shown above, will induce a secondary immune response through the activation of T cells. Additionally, D2C7-IT targets both EGFRwt and mutant EGFRvIII proteins. Since *EGFR* modifications and/or focal amplification has been identified in 57% of GBM patients [6] and 67% of patients with EGFR amplification also express EGFRvIII [45], D2C7-IT will likely demonstrate clinical efficacy and will impact GBM therapy. Further lack of randomization and use of potentially outdated historical controls could have skewed the interpretation of results in early phase rindopepimut trials. Nonetheless, patient dropout could be an issue in randomized open-label or double-blinded studies, and we also believe that treating patients with a sham saline CED infusion would be unethical. Therefore potential over interpretation of D2C7-IT efficacy in phase II clinical trials can be avoided by estimating the overall survival of D2C7-IT patients as compared with a matched historical control group (patients who would have been eligible for the D2C7-IT study if the study had been available at the time of their disease progression).

## Conclusions

In summary for many cancer types, combination therapies with checkpoint inhibitors are widely regarded as the future of modern oncology. Following this strategy, we investigated the antitumor efficacy of an EGFRwt and EGFRvIII dual-specific immunotoxin, D2C7-IT, and checkpoint inhibitor combinations in mouse models of malignant glioma. Our data indicate that in addition to direct tumor cell killing, D2C7-IT induces T cell-mediated antitumor immune response which can be augmented by αPD-L1/αPD-1 combinations. The D2C7-IT and checkpoint inhibitor combination elicit a sustained antitumor immune response that provides long-term protection against tumor re-challenge. Our data support the development of D2C7-IT+αPD-L1/αPD-1 combination therapy in the clinic to improve glioblastoma patient survival. Based on our studies in two syngeneic immunocompetent murine glioma models, we plan to conduct a D2C7-IT and αPD-L1 blockade combination clinical trial in patients with recurrent glioblastoma.

## Additional files

Additional file 1: **Figure** **S1.** D2C7 epitope comparison between human (h) and mouse (ms) EGFR sequence. The diagram shows sequence alignment between the human and mouse EGFR protein with the mismatched amino acids highlighted. **Figure S2.** Toxicity assessment of D2C7-IT in C57BL/6J mice bearing intracranial CT2A-dmEGFRvIII-Luc tumors. Different doses (0.03-1 μg) of D2C7-IT were delivered intracranially by CED over a 3-day period to C57BL/6J mice (N = 8-9 mice/group) implanted with CT2A-dmEGFRvIII-Luc tumors. Animals were monitored for toxicity related death. Data are expressed as percentage of mice surviving versus time. **Figure S3.** Specificity assessment of D2C7-IT in C57BL/6J mice bearing intracranial CT2A-dmEGFRvIII-Luc (**A**) or parental CT2A (**B**) tumors. A total dose of 0.1 μg of D2C7-IT or control P588-IT were delivered intracranially by CED over a 3-day period to C57BL/6J mice (N = 10 mice/group) implanted with CT2A-dmEGFRvIII-Luc (**A**) or parental CT2A tumors (**B**). Animals were monitored for survival. Treatment schedule, survival curves, median survival. And p-values generated from the generalized Wilcoxon test are provided. **Figure S4.** Flow cytometric analysis of immune checkpoint molecule expression on T cells and tumor cells. Intracranial CT2A-dmEGFRvIII-Luc (**A-D**) and SMA560-dmEGFRvIII-Luc (**E-H**) tumors were analyzed for the expression of PD-1 on CD4+ and CD8+ T cells, FoxP3 on CD4+CD25+ T cells and PD-L1 on tumor cells. **Figure S5.** Immunofluorescence and immunohistochemistry analysis of checkpoint molecule expression in orthotopic glioma models. Tissue sections from intracranial CT2A-dmEGFRvIII-Luc (**A**) and SMA560-dmEGFRvIII-Luc (**B**) tumors were analyzed for the expression of PD-1 and FoxP3 on CD4+ and CD8+ T cells and PD-L1 on tumor cells. **Figure S6.** Anti-tumor efficacy of D2C7-IT and immune checkpoint inhibitor combinations in orthotopic glioma models. (**A-D**) Survival curve and median survival estimate data are shown for C57BL/6J mice bearing intracranial CT-2A-dmEGFRvIII-Luc tumors treated with vehicle control, D2C7-IT, αPD-L1 (**A**), αTim-3 (**B**), αLag-3 (**C**), and αCD73 (**D**) mono or combination therapies. The p-values generated from the generalized Wilcoxon test are provided for all studies and are not adjusted for multiple testing. **Figure S7.** Comparison of CD4+ T cells:Treg and CD8+ T cells:Treg ratio in orthotopic CT2A-dmEGFRvIII-Luc tumors after D2C7-IT, αCTLA-4, or αPD-1 mono and combination therapies. Intracranial CT2A-dmEGFRvIII-Luc (N=5/6 mice/group) tumors were investigated for the presence of CD4+CD25+FOXP3+ Tregs by flow cytometric analysis. Panels (**A**) and (**B**) represent CD4+ T cells:Treg and CD8+ T cells:Treg ratio after D2C7-IT or αPD-1 mono and combination therapies. Panels (**C**) and (**D**) represent CD4+ T cells:Treg and CD8+ T cells:Treg ratio after D2C7-IT or αCTLA-4 mono and combination therapies. (PPTX 6598 kb)

Additional file 2: Materials and Methods. (DOCX 28 kb)

## Abbreviations

ADCC: Antibody-dependent cellular cytotoxicity
BLI: Bioluminescence imaging
CED: Convection-enhanced delivery
CTLA-4: Cytotoxic T-lymphocyte-associated protein 4
dmEGFRvIII: D2C7 (d)-mouse (m)-EGFRvIII
EGFRvIII: mutant epidermal growth factor receptor variant III
EGFRwt: wild-type epidermal growth factor receptor
h: human
IC: Intracranial
IT: Immunotoxin
LAG-3: Lymphocyte-activation gene 3
Luc: Luciferase
m: mouse
mAb: monoclonal antibody
MS: Median survival
MSA: Mouse serum albumin
MTD: Maximum tolerated dose
NK Cells: Natural Killer Cells
ORR: Objective response rate
OS: Overall survival
PBS: Phosphate-buffered saline
PD-1: Programmed cell death protein 1
PD-L1: Programmed death-ligand 1
PE: *Pseudomonas* exotoxin A
TIGIT: T cell immunoreceptor with Ig and ITIM domains
TIM-3: T-cell immunoglobulin and mucin-domain containing-3
Tregs: regulatory T cells
WHO: World Health Organization
