# Supplementary material for: Improved efficacy against malignant brain tumors with EGFRwt/EGFRvIII targeting immunotoxin and checkpoint inhibitor combinations
Source: J Immunother Cancer. 2019 May 29;7:142. doi: 10.1186/s40425-019-0614-0 (PMC6542114; doi:10.1186/s40425-019-0614-0)
Supplement: Supplementary file 1 — Figure S1. D2C7 epitope comparison between human (h) and mouse (ms) EGFR sequence. The diagram shows sequence alignment between the human and mouse EGFR protein with the mismatched amino acids highlighted. Figure S2. Toxicity assessment of D2C7-IT in C57BL/6J mice bearing intracranial CT2A-dmEGFRvIII-Luc tumors. Different doses (0.03-1 μg) of D2C7-IT were delivered intracranially by CED over a 3-day period to C57BL/6J mice (N = 8-9 mice/group) implanted with CT2A-dmEGFRvIII-Luc tumors. Animals were monitored for toxicity related death. Data are expressed as percentage of mice surviving versus time. Figure S3. Specificity assessment of D2C7-IT in C57BL/6J mice bearing intracranial CT2A-dmEGFRvIII-Luc (A) or parental CT2A (B) tumors. A total dose of 0.1 μg of D2C7-IT or control P588-IT were delivered intracranially by CED over a 3-day period to C57BL/6J mice (N = 10 mice/group) implanted with CT2A-dmEGFRvIII-Luc (A) or parental CT2A tumors (B). Animals were monitored for survival. Treatment schedule, survival curves, median survival. And p-values generated from the generalized Wilcoxon test are provided. Figure S4. Flow cytometric analysis of immune checkpoint molecule expression on T cells and tumor cells. Intracranial CT2A-dmEGFRvIII-Luc (A-D) and SMA560-dmEGFRvIII-Luc (E-H) tumors were analyzed for the expression of PD-1 on CD4+ and CD8+ T cells, FoxP3 on CD4+CD25+ T cells and PD-L1 on tumor cells. Figure S5. Immunofluorescence and immunohistochemistry analysis of checkpoint molecule expression in orthotopic glioma models. Tissue sections from intracranial CT2A-dmEGFRvIII-Luc (A) and SMA560-dmEGFRvIII-Luc (B) tumors were analyzed for the expression of PD-1 and FoxP3 on CD4+ and CD8+ T cells and PD-L1 on tumor cells. Figure S6. Anti-tumor efficacy of D2C7-IT and immune checkpoint inhibitor combinations in orthotopic glioma models. (A-D) Survival curve and median survival estimate data are shown for C57BL/6J mice bearing intracranial CT-2A-dmEGFRvIII-Luc tumors treat [file 40425_2019_614_MOESM1_ESM.pptx]

## Slide 1
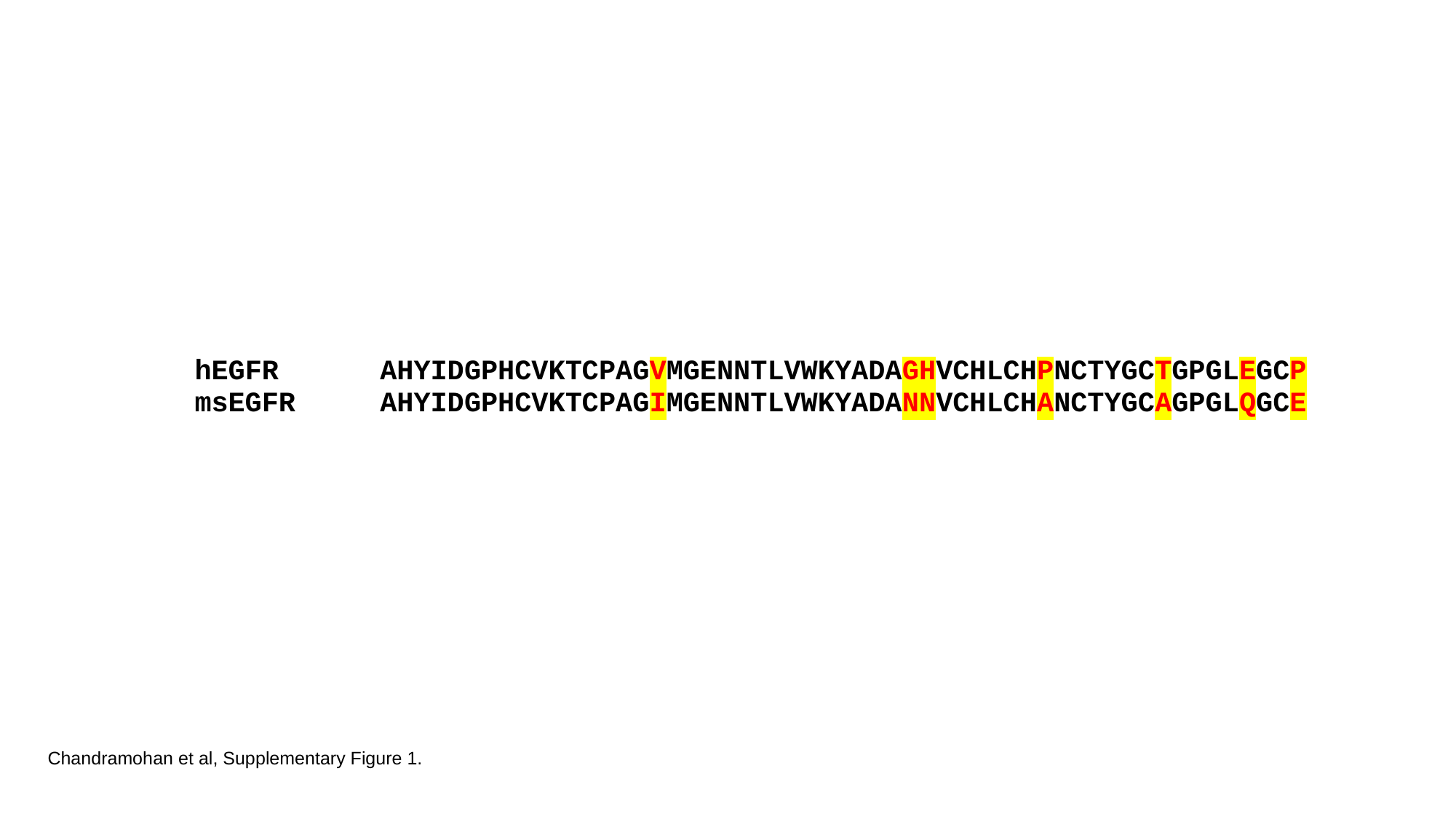

Chandramohan et al, Supplementary Figure 1.

## Slide 2
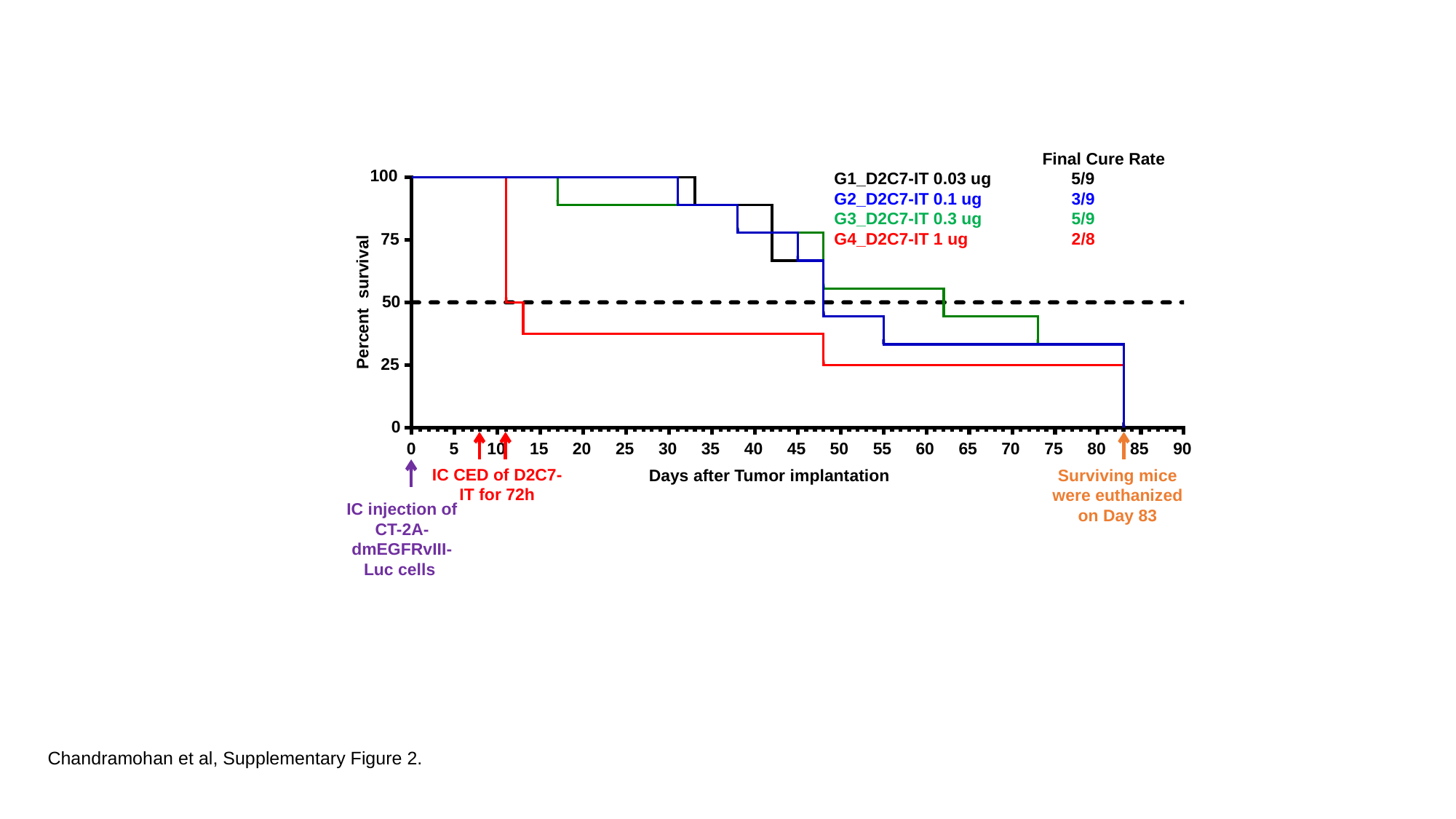

Final Cure Rate
G1_D2C7-IT 0.03 ug 5/9
G2_D2C7-IT 0.1 ug 3/9
G3_D2C7-IT 0.3 ug 5/9
G4_D2C7-IT 1 ug 2/8
100
75
50
Percent survival
25
0
0
5
10
15
20
25
30
35
40
45
50
55
60
65
70
75
80
85
90
IC CED of D2C7-IT for 72h
Days after Tumor implantation
Surviving mice were euthanized on Day 83
IC injection of CT-2A-dmEGFRvIII-Luc cells
Chandramohan et al, Supplementary Figure 2.

## Slide 3
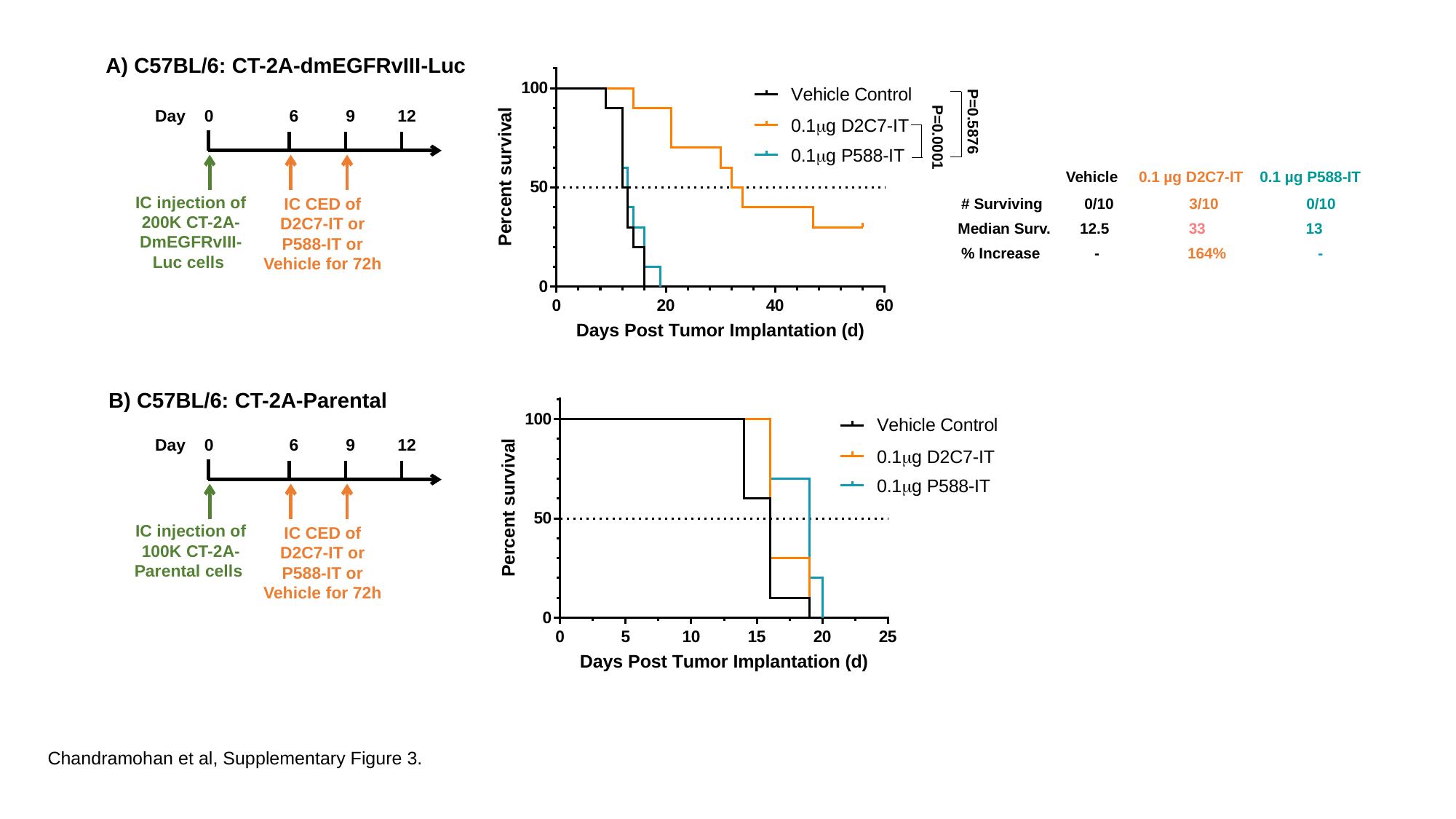

A) C57BL/6: CT-2A-dmEGFRvIII-Luc
P=0.5876
P=0.0001
Day 0 6 9 12
IC injection of 200K CT-2A-DmEGFRvIII-Luc cells
IC CED of D2C7-IT or P588-IT or Vehicle for 72h
 Vehicle 0.1 µg D2C7-IT 0.1 µg P588-IT
# Surviving 0/10 3/10 0/10
Median Surv. 12.5 33 13
% Increase - 164% -
B) C57BL/6: CT-2A-Parental
Day 0 6 9 12
IC injection of 100K CT-2A-Parental cells
IC CED of D2C7-IT or P588-IT or Vehicle for 72h
Chandramohan et al, Supplementary Figure 3.

## Slide 4
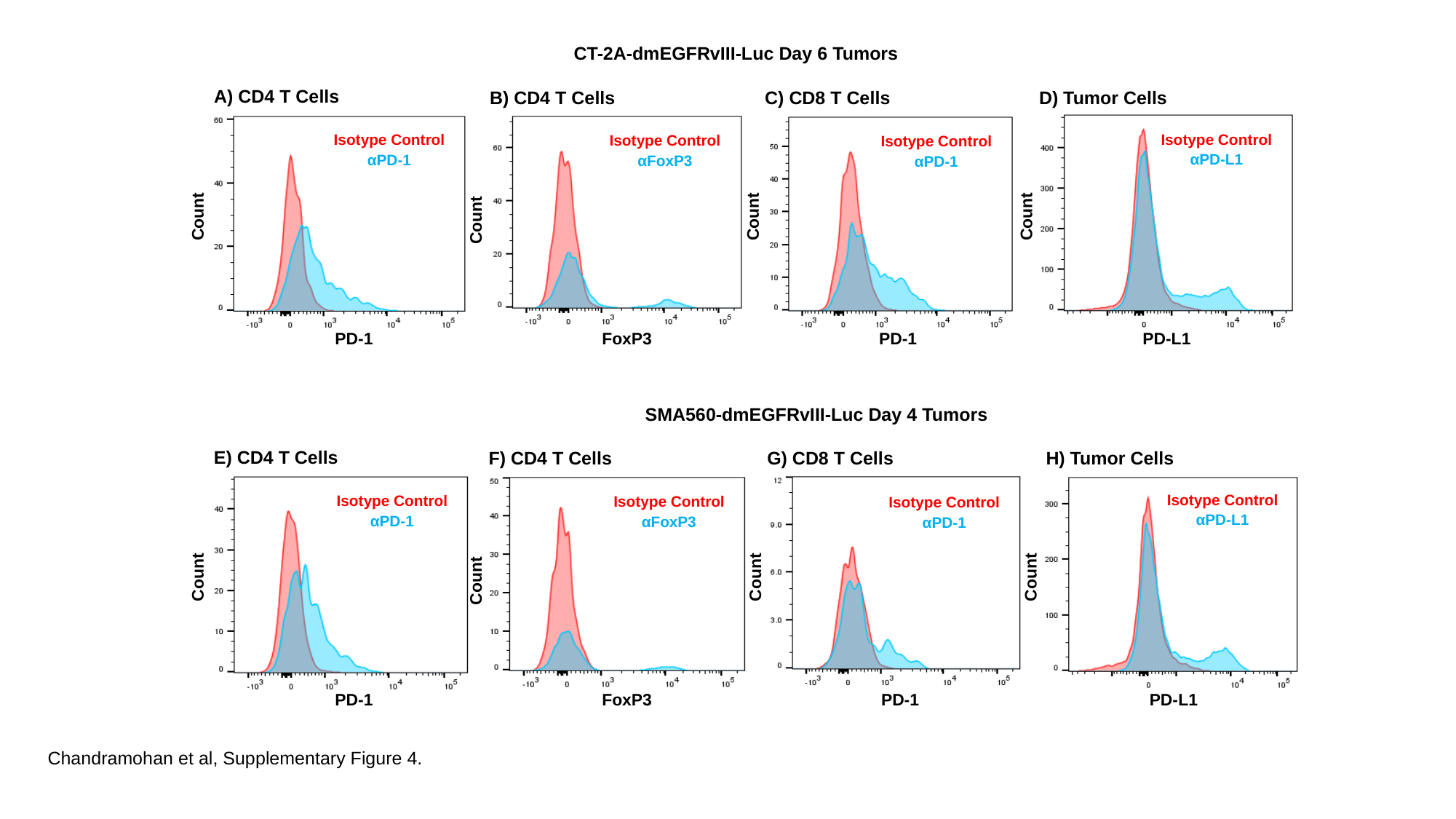

CT-2A-dmEGFRvIII-Luc Day 6 Tumors
A) CD4 T Cells
Count
PD-1
B) CD4 T Cells
Count
FoxP3
C) CD8 T Cells
Count
PD-1
D) Tumor Cells
Count
PD-L1
Isotype Control
αPD-L1
Isotype Control
αPD-1
Isotype Control
αFoxP3
Isotype Control
αPD-1
SMA560-dmEGFRvIII-Luc Day 4 Tumors
E) CD4 T Cells
Count
PD-1
F) CD4 T Cells
Count
FoxP3
G) CD8 T Cells
Count
PD-1
H) Tumor Cells
Count
PD-L1
Isotype Control
αPD-L1
Isotype Control
αPD-1
Isotype Control
αFoxP3
Isotype Control
αPD-1
Chandramohan et al, Supplementary Figure 4.

## Slide 5
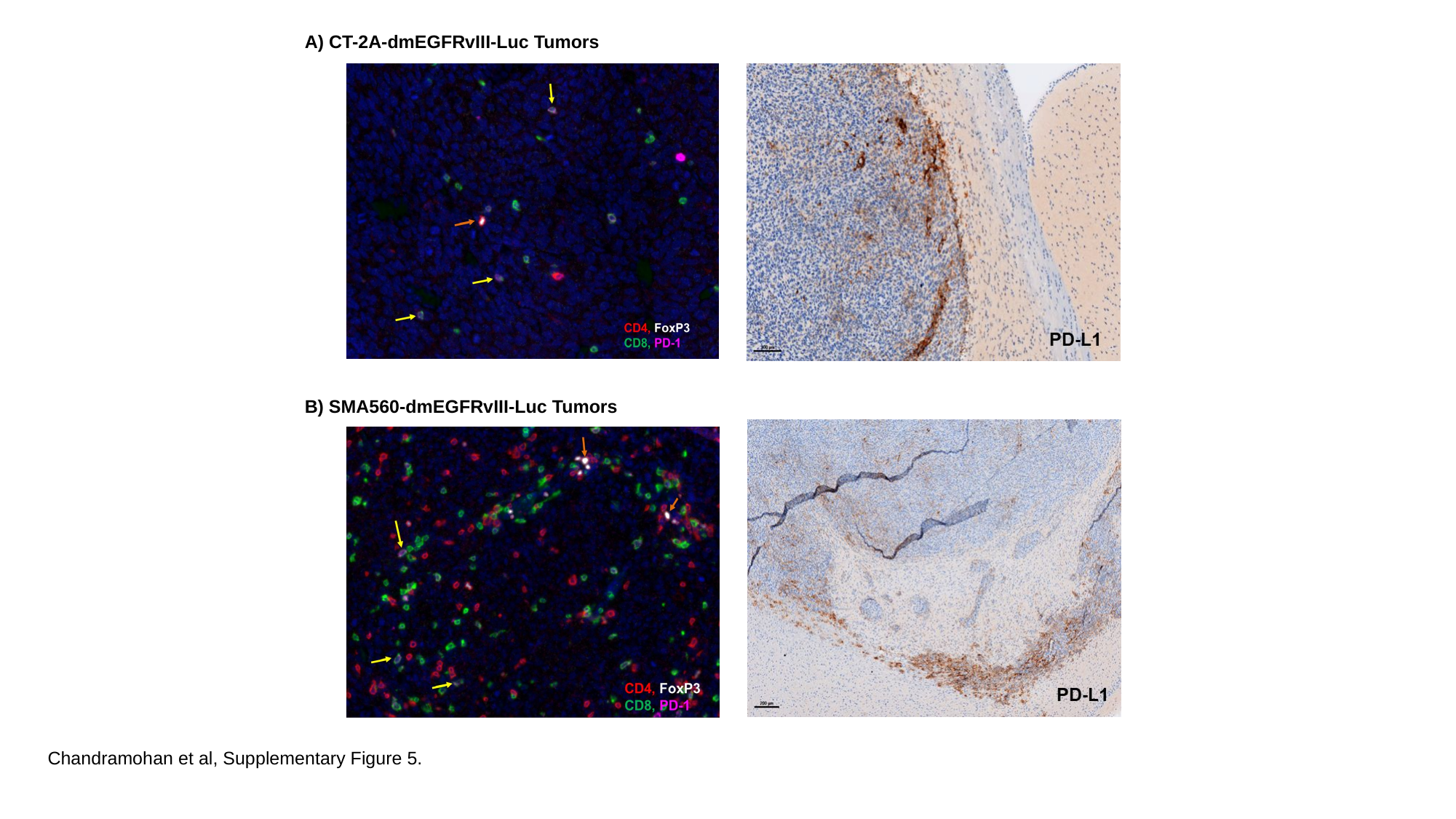

A) CT-2A-dmEGFRvIII-Luc Tumors
B) SMA560-dmEGFRvIII-Luc Tumors
Chandramohan et al, Supplementary Figure 5.

## Slide 6
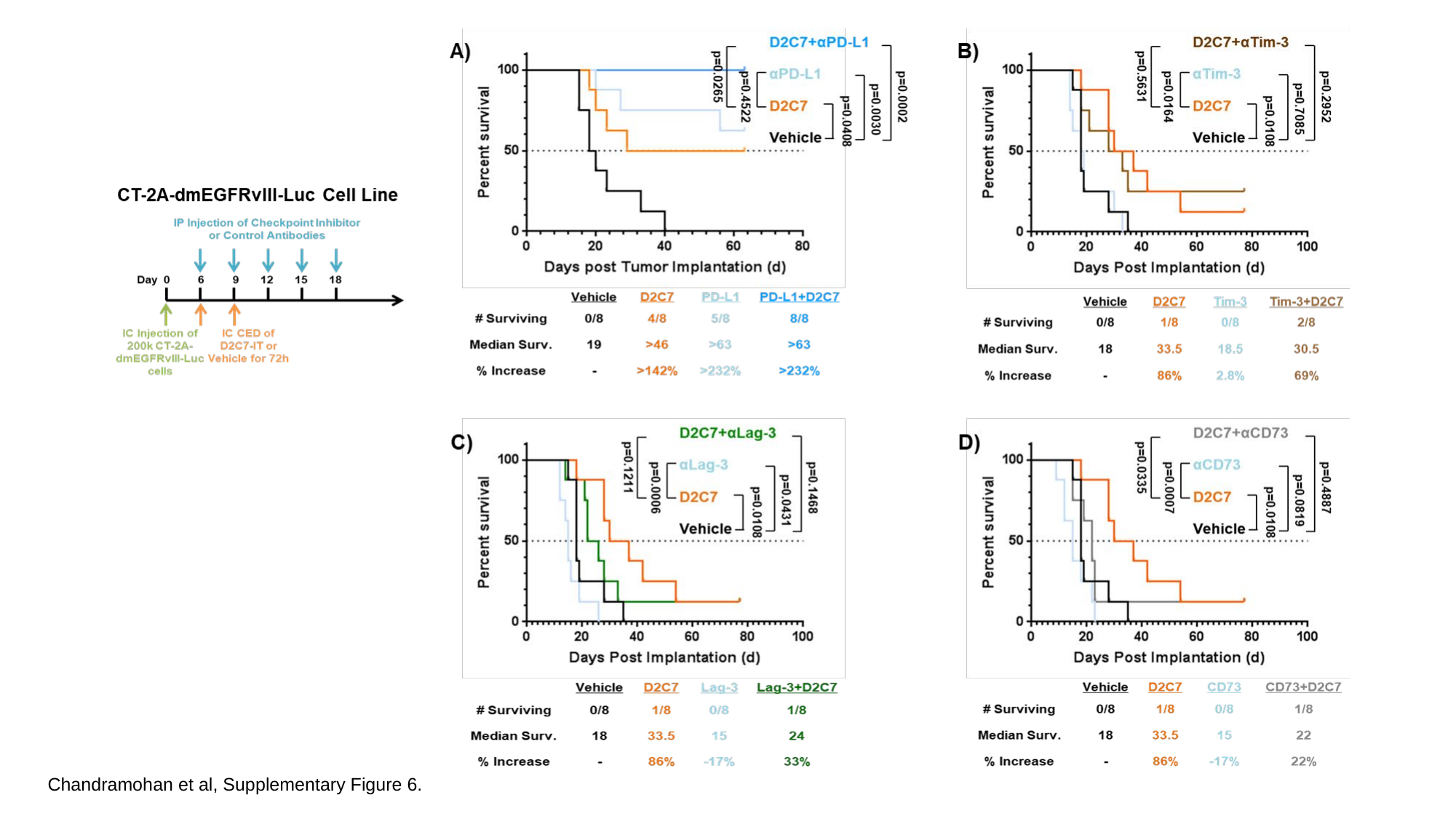

Chandramohan et al, Supplementary Figure 6.

## Slide 7
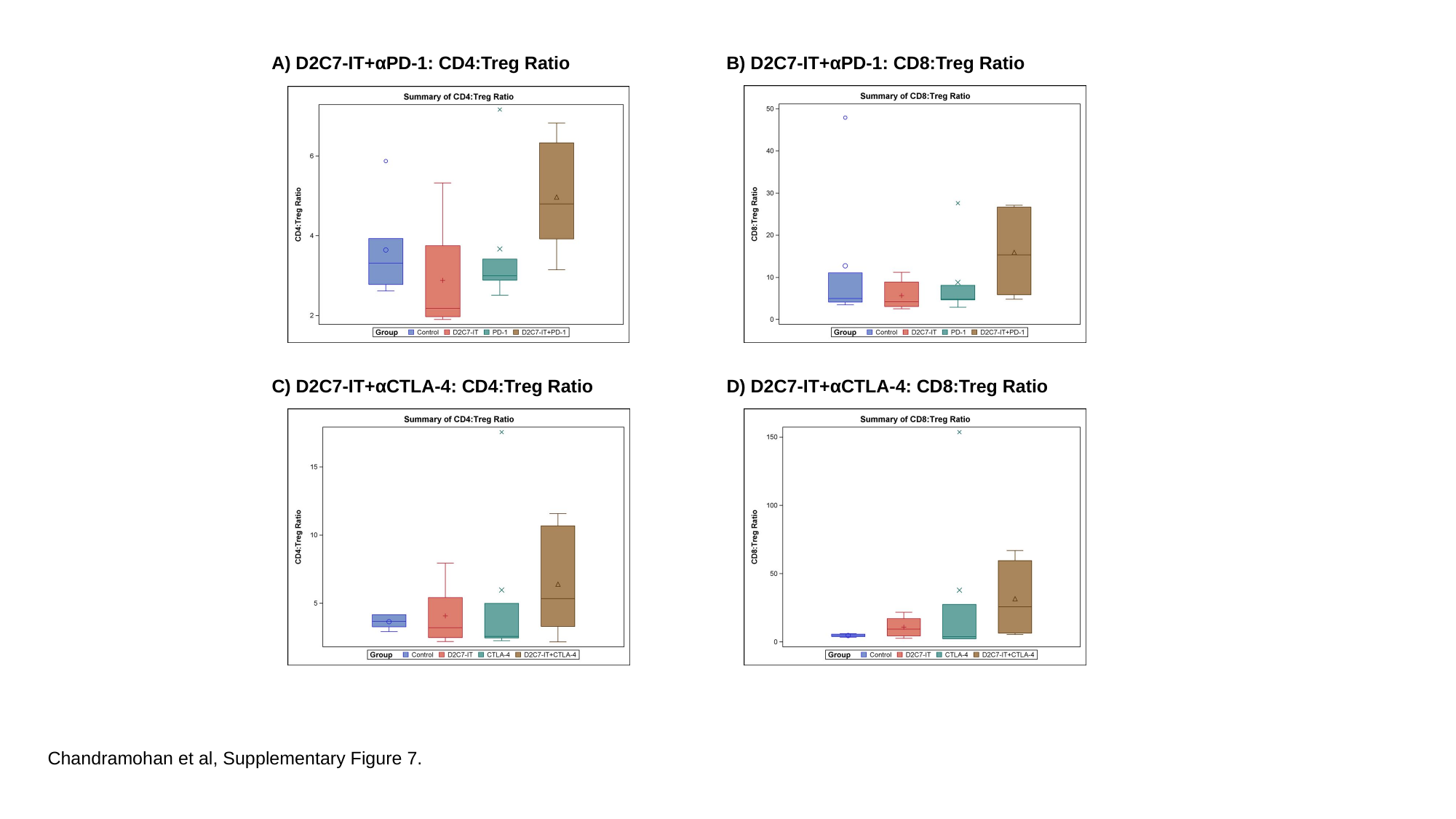

A) D2C7-IT+αPD-1: CD4:Treg Ratio
B) D2C7-IT+αPD-1: CD8:Treg Ratio
C) D2C7-IT+αCTLA-4: CD4:Treg Ratio
D) D2C7-IT+αCTLA-4: CD8:Treg Ratio
Chandramohan et al, Supplementary Figure 7.
