# Supplementary material for: Improved efficacy against malignant brain tumors with EGFRwt/EGFRvIII targeting immunotoxin and checkpoint inhibitor combinations
Source: J Immunother Cancer. 2019 May 29;7:142. doi: 10.1186/s40425-019-0614-0 (PMC6542114; doi:10.1186/s40425-019-0614-0)
Supplement: Supplementary file 2 — Materials and Methods. (DOCX 28 kb) [file 40425_2019_614_MOESM2_ESM.docx]

**Supplementary Materials and Methods**

**Cell lines:** Mouse brain tumor cell lines CT-2A and SMA560 were cultured in an incubator at 37˚C, 5% CO_2_, and passaged at confluence with Accutase Cell Detachment Solution (BD Biosciences). The SMA560 cell line was maintained in complete zinc option (ZO)-10% fetal bovine serum (FBS; Improved Modified Eagle Medium ZO [Richter's Modification, Cat.No.10373-017] liquid; Thermo Fisher Scientific). The CT-2A cell line was cultured in DMEM-high glucose media (Thermo Fisher Scientific) supplied with 10% FBS. All cell lines were authenticated by whole-exome sequencing, tested for rodent pathogens, and the cell culture supernatants were tested for mycoplasma infection. All cell lines were maintained in culture for 10-12 passages after thawing.

**Establishment of D2C7-IT target cell lines:** The chimeric DNA fragment designated D2C7 (d)-mouse (m)-EGFRvIII (dmEGFRvIII) was ligated into the pLXIN retroviral vector (Clontech Laboratories). Mouse astrocytoma cell lines CT-2A and SMA560 were transduced with the dmEGFRvIII retroviral particles and luciferase (Luc) lentiviral particles, and stable cell lines (CT-2A-dmEGFRvIII-Luc and SMA560-dmEGFRvIII-Luc) were established upon G418 and hygromycin selection.

**Preparation of recombinant immunotoxins:** D2C7-IT was expressed under the control of T7 promoter in E. coli BL21 (λ DE3) (Stratagene). D2C7-IT accumulating in inclusion bodies was reduced, refolded, and further purified as monomers by ion exchange and size-exclusion chromatography to greater than 95% purity as described previously (1). The purified immunotoxin was then subjected to endotoxin removal with ActiClean Etox resin (Sterogene).

***In vitro* cytotoxicity assay:** Cytotoxicity of the D2C7-IT on CT-2A-dmEGFRvIII-Luc and SMA560-dmEGFRvIII-Luc was assessed by using the cell proliferation reagent WST-1 (Roche). Cells were seeded in 96-well plates at a density of 1×10^4^ cells per well in 100 µL of complete ZO or DMEM medium 24 h before the assay. D2C7-IT was serially diluted to achieve a final concentration of 0.001 to 1000 ng/mL in PBS containing 0.2% bovine serum albumin. Plates were incubated for an additional 48 h at 37°C and then treated with 10 µl/well of WST-1 and incubated for an additional 1-2 h at 37°C. Cell death was determined by measuring absorbance at 440 nm. The cytotoxic activity was defined by IC_50_, which was the toxin concentration that caused cell death in 50% of the cells. All experiments were repeated at least three times.

**Tumor tissue processing:** Orthotopic tumor-bearing mice were euthanized with Isoflurane. After euthanasia, animals were perfused through the left ventricle with PBS, brains were harvested, and tumor-bearing right hemispheres were collected. All tissues were cut into small pieces and placed in digestion solution containing 1.5mg/ml of Collagenase A (Roche) and 0.4mg/ml DNaseI (Roche) in HBSS plus 5% fetal bovine serum and 10mM HEPES. Tissues were incubated at 37°C for 45 minutes with gentle vortexing every 10-15 minutes. Upon completion of digestion, 25ml of PBS was added, and the samples were vortexed at maximal speed for 30 seconds. The resulting cell suspensions were strained through a 70um cell strainer, pelleted, centrifuged through a continuous 30% Percoll gradient to remove myelin and other cell debris, and re-suspended in PBS with 1% BSA, and counted with trypan blue solution.

**Flow cytometry:** Flow cytometry analysis was performed with the D2C7 mAb. Briefly, 1×10^6^ cells (CT-2A-dmEGFRvIII-Luc and SMA560-dmEGFRvIII-Luc) were suspended in 500 µL of phosphate-buffered saline pH 7.4 (PBS) (Thermo Fisher Scientifi) containing 5% FBS (Thermo Fisher Scientifi) (5% FBS/PBS). The IgG1-AF488/-APC or D2C7-AF488/-APC was added to the cells at a concentration of 5 µg/mL, and the samples were incubated for 40 min at 4ºC. Stained cells were analyzed on a BD FACSCalibur instrument equipped with CellQuest software (BD Biosciences).

Tumor cells and total leukocytes isolated from tumor hemispheres were stained as described previously (2). Antibodies used for staining are as follows: CD45 (Biolegend), CD31 (BD Bioscience), CD11b (BD Bioscience), CD11c (BD Bioscience), CD3 (Biolegend), CD4 (Biolegend), CD8 (eBioscience), CD25 (Biolegend), PD-1 (Biolegend), and PD-L1 (Biolegend). Upon completion of surface staining, cells were fixed, and intracellular staining was performed according to the manufacturer's protocol with the Foxp3 Transcription Factor staining buffer set (Thermo Fisher Scientific). After staining, cells were washed and fixed with 0.4% paraformaldehyde in PBS. Data was acquired with a BD LSRII flow cytometer in the Duke Human Vaccine Institute Research Flow Cytometry Facility (Durham, NC) using BD FACSDiva software (BD Bioscience). Compensation was performed on the BD LSRII flow cytometer at the beginning of the experiment. Data were analyzed using Flowjo v10.

**Multiplex immunofluorescence staining:** Serial FFPE sections (5-micron thickness) were stained with CD4 (Cell Signaling Technology), CD8 (Cell Signaling Technology), FoxP3 (Cell Signaling Technology), PD-1 (Cell Signaling Technology), or rabbit isotype control (Cell Signaling Technology) antibodies, and the nuclei were subsequently stained with 4,6-diamidino-2-phenylindole solution (PerkinElmer) using automated IHC techniques on Bond-RXm Processing Module (Leica Microsystems), utilizing the Bond Research Detection kit and Opal fluorophores (PerkinElmer). The sections were coverslipped using Vectashield HardSet Antifade mounting media (Vector Laboratories). The slides were scanned using the Vectra 3.0 System; image analysis was performed using the InForm image analysis software (both PerkinElmer).

**Immunohistochemistry:** FFPE sections (5-micron thickness) were stained with PD-L1 (Cell Signaling Technology) or rabbit isotype control (Cell Signaling Technology) antibodies using automated IHC techniques on Bond-RXm Processing Module, utilizing the Bond Polymer Refine Detection kit (Leica Microsystems).The slides were scanned using the Vectra 3.0 System.

***In vivo* intracranial antitumor studies:** C57BL/6J mice (≈20 g, 6–8 weeks, female) and VM/Dk mice (≈20 g, 6-8 weeks, male, Duke Division of Laboratory Animal Resources) were anesthetized and mounted onto a stereotactic frame. The anterior cranial region was shaved, and a pilot hole or an incision ≈1 cm in length was made in the skin over the skull at coordinates 2.0 mm left lateral of the sagittal and 0.5 mm anterior to the bregma using a Dremel 105 Engraving Cutter or manually with a needle. A 25 or 27-gauge disposable needle attached to a 25-μl Hamilton syringe was inserted vertically to a depth of 2.5 mm or 3.3 mm from the dura mater. A total of 1.5-2x10^5^ CT-2A-dmEGFRvIII-Luc mouse glioma cells or 3x10^4^ SMA560-dmEGFRvIII-Luc mouse glioma cells were injected in 5 μl of PBS containing 3% methylcellulose (Sigma) at a rate of 3.33 μL per minute using an automated injector.

For the D2C7-IT monotherapy or combination therapy studies, mice underwent bioluminescence imaging (PerkinElmer) and were randomized into different treatment groups (10 mice/group) by total flux one day before D2C7-IT infusion. Post-implantation of CT-2A-dmEGFRvIII-Luc (day=6) and SMA560-dmEGFRvIII-Luc (day=4) cells, D2C7-IT (0.1 or 0.3 μg total dose in 2% PBS-MSA) was infused by convection-enhanced delivery (CED) at a rate of 0.5 μl/h for 72 h via alzet osmotic minipumps (Durect Corporation#1007D). The control mice were handled in the same manner but treated with 2% PBS-MSA.

For the CD4 and CD8 T cell depletion studies, mice were randomized into different treatment groups (10 mice/group) based on their weight measurements before tumor implantation. The CT-2A-dmEGFRvIII-Luc and SMA560-dmEGFRvIII-Luc cells were implanted and treated as specified above by CED with a total dose of 0.3 μg D2C7-IT. Five doses (250 ug/dose) of rat IgG2b isotype control antibody (Clone LTF-2, Bio-X-Cell), αCD4 antibody (Clone GK1.5, Bio-X-Cell), or αCD8 antibody (Clone 2.43, Bio-X-Cell) were administered intraperitoneally post-implantation of CT-2A-dmEGFRvIII-Luc cells (days 2, 6, 9, 12, and 15) and SMA560-dmEGFRvIII-Luc cells (days 0, 4, 7, 10, and 13). Near total depletion of CD4+ and CD8+ T cells after αCD4 or αCD8 treatment was confirmed by the analysis of spleen by flow cytometry.

For the αCTLA-4, αPD-1, αPD-L1, αTim-3, αLag-3, or αCD-73, efficacy studies with CT-2A-dmEGFRvIII-Luc and SMA560-dmEGFRvIII-Luc cells, mice were randomized and treated with a total dose of 0.1 μg D2C7-IT/vehicle control by CED as described above. Post-implantation of CT-2A-dmEGFRvIII-Luc (αCTLA-4/αPD-1: days 6, 9, 12, 15, and 18 and αPD-1/αPD-L1/αTim-3/αLag-3/αCD-73: days 3, 6, 9, 12, and 15) and SMA560-dmEGFRvIII-Luc cells (days 4, 7, 10, 13, and 16), five doses of 250 µg/dose of rat IgG2a isotype control antibody (Clone 2A3, Bio-X-Cell), αPD-1 antibody, αPD-L1 antibody (Clone 10F.9G2, Bio-X-Cell), αTim-3 antibody (Clone RMT3-23, Bio-X-Cell), αLag-3 antibody (Clone C9B7W, Bio-X-Cell), αCD-73 antibody (Clone TY/23, Bio-X-Cell) or 100 µg/dose αCTLA-4 antibody were delivered by intraperitoneal injections.

The antitumor response of intracranial (ic) tumors to treatment was assessed by the percentage increase in time to a specific neurologic endpoint (seizure activity, repetitive circling, or other subtle changes such as a decrease in appetite) or death. Animals were observed twice daily for signs of distress or development of neurologic symptoms, at which time, the mice were euthanized. All experiments were repeated at least two times. Pairwise differences of survival curves were assessed using the generalized Wilcoxon test.

Mice from the efficacy studies surviving symptom-free for >70 days post initial tumor implantation were rechallenged with 1.5x10^5^ CT-2A-dmEGFRvIII-Luc, 2x10^5^ CT-2A parental, or 3x10^4^ SMA560 parental cells on the contralateral hemisphere using the coordinates described earlier. A total of five C57BL/6J or VM/Dk mice were used as controls. Animals were observed twice daily for signs of distress or development of neurologic symptoms, at which time, they were euthanized.

**Reference**

1. Chandramohan V, Bao X, Keir ST, Pegram CN, Szafranski SE, Piao H, et al. Construction of an immunotoxin, D2C7-(scdsFv)-PE38KDEL, targeting EGFRwt and EGFRvIII for brain tumor therapy. Clin Cancer Res. 2013;19(17):4717-27.

2. Yu YR, O'Koren EG, Hotten DF, Kan MJ, Kopin D, Nelson ER, et al. A Protocol for the Comprehensive Flow Cytometric Analysis of Immune Cells in Normal and Inflamed Murine Non-Lymphoid Tissues. PLoS One. 2016;11(3):e0150606.
